# Recent Advances in Nano-Enabled Seed Treatment Strategies for Sustainable Agriculture: Challenges, Risk Assessment, and Future Perspectives

**DOI:** 10.1007/s40820-023-01025-5

**Published:** 2023-02-16

**Authors:** Amruta Shelar, Shivraj Hariram Nile, Ajay Vikram Singh, Dirk Rothenstein, Joachim Bill, Jianbo Xiao, Manohar Chaskar, Guoyin Kai, Rajendra Patil

**Affiliations:** 1 Department of Technology, Savitribai Phule Pune University, Pune, Maharashtra 411007 India; 2 Zhejiang Provincial International S&T Cooperation Base for Active Ingredients of Medicinal and Edible Plants and Health, School of Pharmaceutical Science, Jinhua Academy, Zhejiang Chinese Medical University, Hangzhou, 310053 Zhejiang People’s Republic of China; 3 Department of Chemical and Product Safety, German Federal Institute for Risk Assessment (BfR), Max-Dohrn-Strasse, 10589 Berlin, Germany; 4 Institute for Materials Science, University of Stuttgart, 70569 Stuttgart, Germany; 5 International Research Center for Food Nutrition and Safety, Jiangsu University, Zhenjiang, 212013 People’s Republic of China; 6 Faculty of Science and Technology, Savitribai Phule Pune University, Pune, Maharashtra 411007 India; 7 Department of Biotechnology, Savitribai Phule Pune University, Pune, Maharashtra 411007 India

**Keywords:** Agro seeds, Environmental seed stressors, Nanoagrochemicals, Toxicological implications, Risk regulations

## Abstract

Agro seeds are vulnerable to environmental stressors, adversely affecting seed vigor, crop growth, and crop productivity. Different agrochemical-based seed treatments enhance seed germination, but they can also cause damage to the environment; therefore, sustainable technologies such as nano-based agrochemicals are urgently needed. Nanoagrochemicals can reduce the dose-dependent toxicity of seed treatment, thereby improving seed viability and ensuring the controlled release of nanoagrochemical active ingredients However, the applications of nanoagrochemicals to plants in the field raise concerns about nanomaterial safety, exposure levels, and toxicological implications to the environment and human health. In the present comprehensive review, the development, scope, challenges, and risk assessments of nanoagrochemicals on seed treatment are discussed. Moreover, the implementation obstacles for nanoagrochemicals use in seed treatments, their commercialization potential, and the need for policy regulations to assess possible risks are also discussed. Based on our knowledge, this is the first time that we have presented legendary literature to readers in order to help them gain a deeper understanding of upcoming nanotechnologies that may enable the development of future generation seed treatment agrochemical formulations, their scope, and potential risks associated with seed treatment.

## Highlights

- Novel insights on recent advances in nanotechnology-based agro seed treatment formulations.
- Details on reducing the environmental impact of seed treatment by using nanoagrochemicals.
- Applications of potential of nanopesticides and nanofertilizers for sustainable seed treatments.
- Described scope of possible next-generation nanomaterials for seed treatment formulations with associated challenges and risks assessment methodologies.

## Graphical abstract

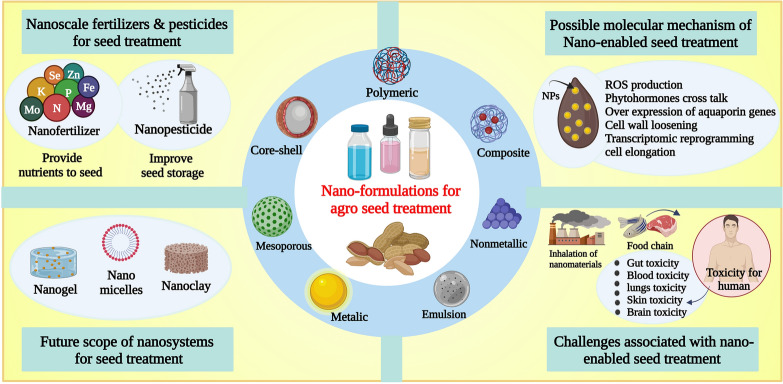

## Introduction

The agricultural industry plays a significant role in developing economies and provides food for a rapidly growing world population of nearly 7.5 billion people [1, 2]. Since 90% of food crops are grown from seed, the seed is a vital input for sustainable agricultural productivity and production. A healthy agro seed produces healthier, more viable, and vigorous seedlings, contributing to effective agricultural practices. In the current scenario, agriculture faces a wide range of challenges, including changing environmental conditions like salinity, drought, heavy metal accumulation in soil and climate changes etc., which can adversely affect seed germination, seedling development, and ultimately, crop production [3–5]. The quality of seeds may also be reduced by seed-borne diseases or destroyed by insects and other pests [6]. This can lead to abnormal seed dormancy, non-viability, and reduced water absorption, negatively impacting crop production and final yield. Therefore, maintaining the seed quality is crucial for germination, seedling establishment, and crop growth. Agrochemical-based seed treatment can prevent these issues and enhance seed quality by protecting agro seeds from biotic and abiotic stresses. In order to address and prevent various pests, diseases, and nutritional deficiencies, various agrochemicals are used separately and in combination with each other for seed treatments [7–12]. They include fungicides, insecticides, fertilizers, and fertilizer enhancers. Nevertheless, these chemicals are costly, toxic for health, toxic leaching occurs in soil, seed pathogens are showing resistance, and the chemicals reach the water sources like rivers or sea, causing eutrophication, reducing soil fertility, reducing beneficial microbial activity, and altering the pH of the soil. The abundant use of conventional agrochemicals and runoff of their wastes also contributes to nutrient and food chain imbalances in ecosystems, leading to pollution of the environment and soil [13–15]. For this reason, it is imperative to implement sustainable agricultural practices to protect seeds from pests and insects while maintaining the agro-ecosystem. Conventional agrochemicals are discouraged as they are not contributing to sustainable agriculture seed treatment practices due to the issues of leaching, degradation, hydrolysis of agrochemicals. New technologies which are safe and economic, and based on green chemistry approaches are urgently needed to reduce environmental burden on soil [13–15].

**Fig. 1:**
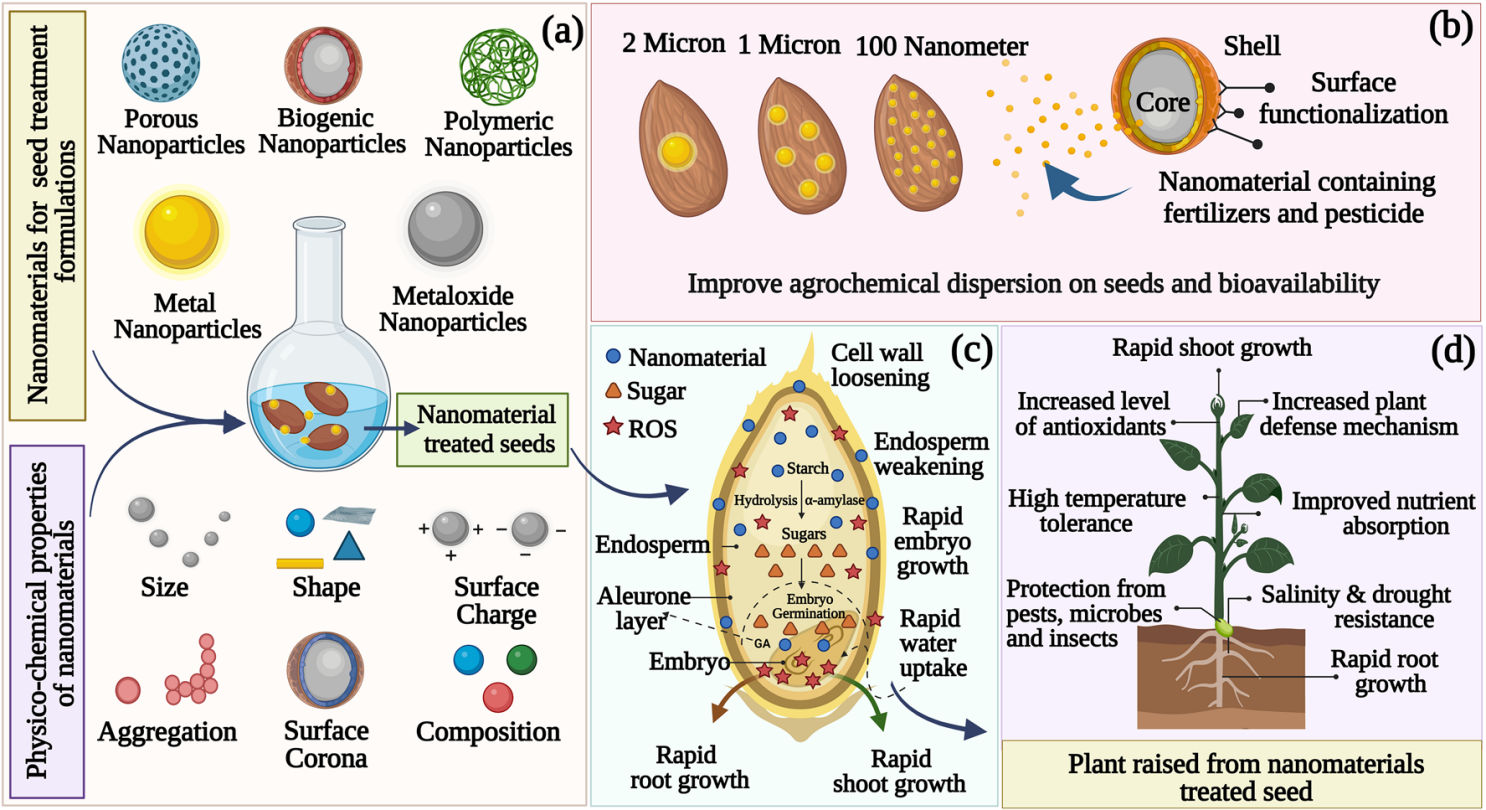
Application of organic–inorganic nanomaterials in seed germination and plant development. **a** Nanoparticles’ properties (such as size, shape, surface charges, composition, and concentration) affecting the seed interaction. **b** Surface-engineered nanoparticles with the desirable properties for seed treatments. **c** Nanoparticles induced seed metabolism. **d** Nanoparticles effect for the improved growth and establishment of plants

New techniques and strategies are constantly evolving to address these pertinent issues with agro seeds. To revolutionize modern agriculture practices, nanomaterial-based products are being introduced. The high surface area-to-volume ratio and novel physicochemical properties of these materials enable them to meet increasing demand due to their high reactivity [16]. Nanotechnology deals with materials with a size ranging from 1 to 100 nm [17–25]. Moreover, by increasing the surface area per mass of a material, a more significant amount of the nanomaterial can contact surrounding materials, influencing its reactivity [26]. The surface area of nanomaterials is much larger than that of similar masses of larger-scale materials [27]. The application of nanotechnology to seed treatment is a relatively new area of research. Nanoagrochemicals for seed treatment can achieve popularity today because they are more effective than conventional agrochemicals, making them economically viable and environmentally friendly. Nanotechnology can significantly contribute to the sustainable development of nanoscale agrochemicals for seed treatment and can enhance the efficiency of agricultural inputs. There has been evidence that nanoparticles increased seed germination and biomass yield on seeds. The nanoparticles have also increased the seed’s resistance to several biotic and abiotic stresses. The biological functions of seeds depend on molecular events. There has been little progress at the molecular level regarding nanoparticle-induced mechanisms and seeds, which is an important step in evaluating potential mechanisms. To understand seed’s underlying mechanisms and responses toward nanoparticles and the changes in gene expression through molecular approaches, it is crucial to understand how seeds respond to nanoparticles. In the last decade, nanomaterials have demonstrated extensive and beneficial chemical interactions with agro seed systems, ranging from seed disease management and yield improvement to environmental safety [17, 28–31]. As shown in Fig. 1a, nanomaterials can be surface engineered to provide desirable properties and functions for particular seed treatment. This will primarily allow them to target the correct locations within the seed or seed coat and offer smart release and delivery strategies (Fig. 1b). Recent studies have found that seeds treated with nanomaterials can activate several genes during germination [32–34]. It has been shown that nanomaterials promote seed germination by forming nanopores in seed coats, introducing reactive oxygen species (ROS), increasing enzyme activity at starch-degrading sites, and introducing ROS to the seed coat (Fig. 1c). A variety of signaling molecules regulate seed germination, including ROS and phytohormones. ROS regulate gene expression and phytohormone signaling, and they maintain a balance between abscisic acid, gibberellins, auxins, and ethylene [8]. In contrast, excessive ROS levels hamper seed germination by causing extensive oxidative damage. Therefore, ROS levels should be controlled spatiotemporally so they can be enclosed in the so-called oxidative window, ensuring proper germination. A significant physiological effect on seed germination appears to be caused by nanoparticles, although the exact mechanism is unknown. Some studies revealed that, ROS can be generated by nanoparticles by triggering the production of •OH radicals. As a result of soaking seeds in nanomaterial containing solutions for a certain period of time, the •OH radicals produced by bound nanoparticles would loosen cell walls, thereby stimulating seedling growth [8]. Using nanoagrochemicals to treat seeds is an efficient method of altering seed metabolism and signaling pathways, which significantly impacts germination and establishment of plant’s overall life cycle (Fig. 1d). By applying nanomaterials to seeds, we can protect them during storage, enhance germination, synchronize germination, improve growth early on, and significantly reduce the amount of pesticides and fertilizers that need to be applied [35].

The literature search has shown that nanomaterials such as silver, gold, copper, palladium, selenium, zinc oxide, magnesium oxide, titanium dioxide, and iron oxide have been proven to promote seed germination and improve crop yields [10, 19–21, 23, 24, 28, 29, 31]. The revolution of next-generation seed agrochemicals will be driven by porous, biogenic, metallic, metal oxide, and polymeric nanomaterials [36–38]. Apart from promoting seeds germination, nanomaterials can serve as seed protectors as well. They can protect seeds from bacteria, fungi, and pests. In some exceptional cases, nanomaterials have been observed to have size- and concentration-dependent toxicity, such as reducing germination rates and causing phytotoxicity to seedlings [39–41]. Nanoparticle toxicity can be reduced by controlling their physicochemical properties, such as size, shape, surface charges, composition, and concentration, which determine their biological response. Using nanoagrochemicals for seed treatment raises the possibility of their release into the ecosystem and soil. Applying these nanoagrochemicals in actual field situations raises concerns about their safety, exposure levels, and toxicological consequences for the environment and human health [42–44]. Depending on their nature and the presence of organic and inorganic constituents, nanoagrochemicals may undergo physical, chemical, and biological transformations once they enter the environment. When the nanoagrochemicals are transformed or aggregated, their stability, reactivity, toxicity, and selectivity may be affected, and their target may be altered [45].

Nanoagrochemicals for seed treatment need to be evaluated more closely to determine their fate in the environment. There is no comprehensive review of seed treatment-specific nanomaterials development, application, safety, and regulation in the literature. The present discussion aims to provide readers with up-to-date information on the latest organic and inorganic nanomaterials used for sustainable seed treatments. Furthermore, this review provides a detailed overview of the safety of nanomaterials in the environment when used for seed treatment. In developing nano-based agrochemicals, some nanomaterials would be excellent candidates for seed-specific treatments and for developing agro products nanoformulations for seed treatment. Nanomaterials-enabling technology and products have the potential to provide one of the most effective and environmentally friendly seed treatment options.

## Nanotechnology Toward Sustainable Seed Treatments

In order to ensure a sustainable future for agriculture, there is an urgent need for sustainable seed treatment practices. Sustainable seed treatment practices ensure profitability, environmental health, social equity, and profitability of existing and future generations. As part of sustainable seed treatment practices, agrochemical usage is to be getting control, since they can contaminate the soil, water, turf, and other vegetation, as well as harm nontarget organisms such as plants, birds, animals, and fish. Agrochemicals which are used for seed treatment are absorbed by surrounding land and water bodies, entering the food chain and accumulating in body [13–15]. As far as their effects on crops are concerned, excessive application of these chemicals generates significant residues. Agrochemical residues contribute to nutrient imbalance and quality reduction of agricultural products. Furthermore, these over use of agrochemicals can adversely affect the environment by causing abnormal climate change, damaging biodiversity, polluting groundwater and soil, destroying natural resources, violating waste management laws, and creating noise pollution and air pollution [13–15]. Therefore, it is necessary to develop sustainable agricultural practices to overcome agrochemical issues. We need smart agrochemicals for sustainable seed treatment in order to achieve this. This approach proposes formulations and products that fulfill the needs for chemicals that provide sustainable nutrient delivery systems that maximize agricultural crop yield and minimize environmental impact [46].

**Fig. 2:**
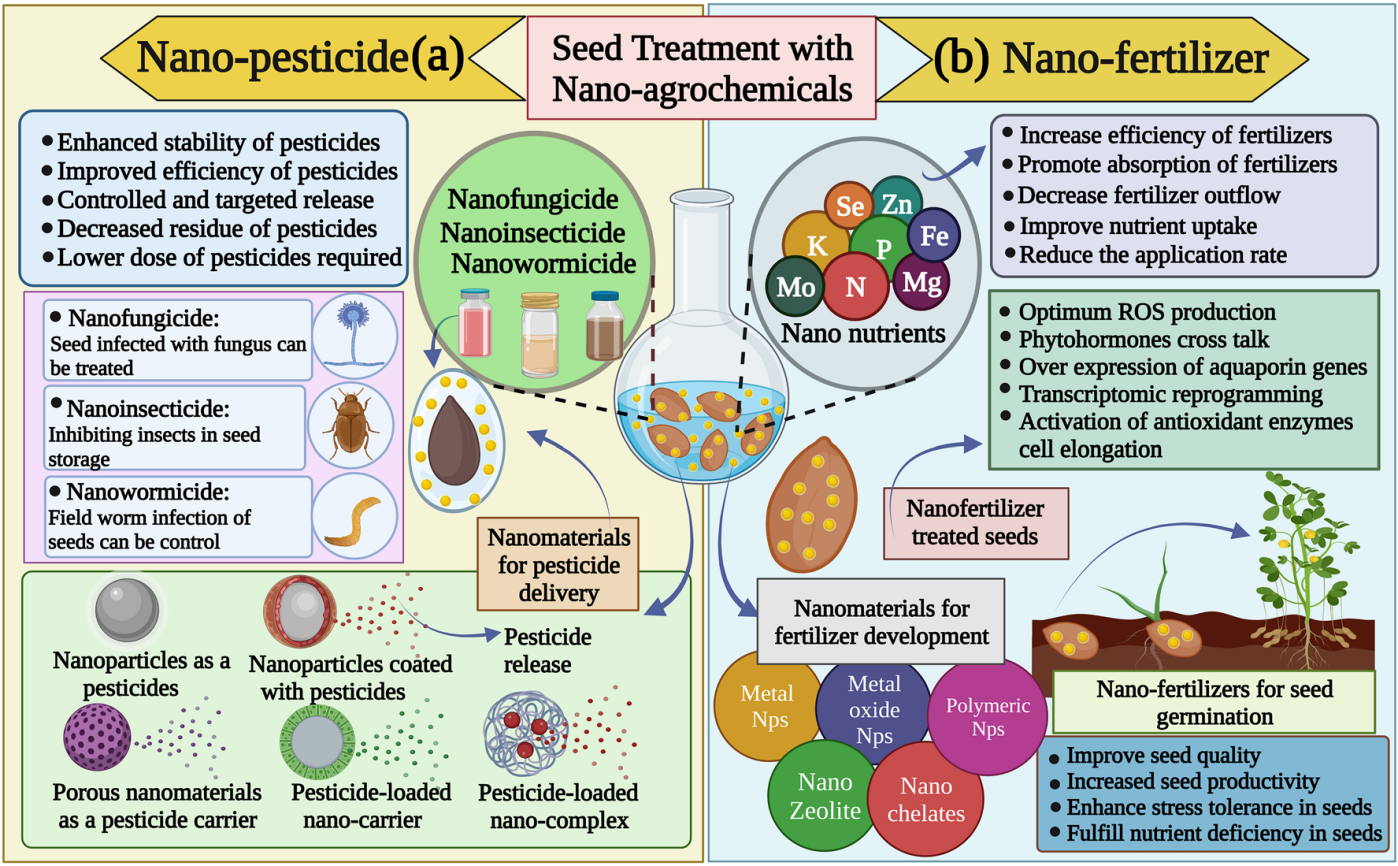
Role of nanoagrochemicals and nanofertilizers in seed treatment. **a** Characteristics of nanopesticides, such as enhanced stability, control, and targeted delivery of agrochemicals, assist the seed in effectively protecting itself from pathogens and pests during germination. **b** Nanofertilizers compositions (Se, Zn, N, P, K, Mo, etc.) providing the nutrient-rich element for enhanced seed protection, enhanced stress tolerance, and fulfilling nutrient deficiency in the soil for the effective seed germination

The use of nanoscale agrochemicals as smart chemicals for seed treatment, such as nanofertilizers, nanopesticides and nanofungicides [47–51], has transformed traditional agriculture practices to become more sustainable and efficient (Fig. 2). Using nanomaterials and nanotechnology in agrochemicals overcomes several disadvantages of conventional agrochemicals, including poor solubility, low bioavailability, easy photolysis, organic solvent pollution and excess toxicity. Nanomaterials have been used successfully in the development of seed treatment over the past few years [52, 53]. The potential applications of nanomaterials for seed treatment can be categorized into active nanoparticles and sustained release nanocarrier systems [54]. The active nanoparticle is a nanoparticle that can cause a biological effect. Active nanoparticles can act as stimulants, anti-pathogens, or both. Active nanoparticles include multi-walled carbon nanotubes that act as an effective agrochemical carrier, metal oxide-based nanoparticles for encouraging germination, nanosilver as antimicrobial agents, nanotitanium oxide for photocatalytic activity and nanosilica to deliver pesticides and fertilizers due to its large surface areas [18, 37, 55, 56]. As depicted in Fig. 2, several metal nanoparticles, including iron, zinc, manganese, selenium, etc., are critical nutrients for seed germination and development. Other studies have used nanopolymers and liposomes as renewable, biodegradable, and environmentally friendly carriers to encapsulate essential oils, pesticides, nutrients, and fertilizers [35, 57–59]. A sustained release nanocarrier encapsulates an active ingredient (biological or synthetic) and delivers this compound continuously over time instead of releasing it all at once.

More than 99.9% of pesticides fail to reach their targets and leave harmful impacts on soil, water, and air health while increasing pathogenic resistance and reducing biodiversity. Using a controlled release nano-system for targeted seed treatment is an efficient route to upgrading and advancing it sustainably. It has been shown that stimuli-response release nano-systems can be observed using photosensitive polymers. In this way, nanocomposite-based stimuli can intelligently react to the stimulation produced by the target or adjacent environment that ultimately triggers the release of agrochemicals to regulate the seed disease effectively. The controlled release nano-system offers several advantages over conventional chemical applications; the controlled release nano-systems allow more efficient delivery of pesticides and fertilizers more quickly into seeds, resulting in a decline in the concentration of agrochemicals used [60, 61]. Various nanoparticle-based products and smart agrochemical delivery systems using nanocomposites are constantly being developed for seed treatment (Table 1). The potential of nanocomposite as a nanofertilizer, nanoherbicide, nanofungicide, and nanoinsecticide for the next-generation treatment for seed treatments offer a variety of advantages, including durability, effectiveness, wettability, good dispersibility, less toxicity, good biodegradability in soil and environment, and photogenerated nature with the least amount of residues compared to conventional chemicals [53].

**Table 1:** An overview of the latest advances in the application of nanomaterials for the treatment of seeds with nanofertilizers and nanopesticides

Type of agrochemicals	Type of nanomaterials used	Type of seeds	Dose concentration	Key features	References
Nanofertilizers	Zinc	Peanut (*A. hypogaea* L.)	100–500 ppm	An improvement in morphological, yield, and biochemical traits	[212]
		Sorghum (*S. bicolor* L.)	6 mg/kg soil	Increased yields and growth, increased nitrogen and potassium uptake, improved grain nutrient profile	[213]
		Maize (*Z. mays* L.)	50–2000 ppm	Improved seed germination, seedling vigor index, biomass, productivity, and zinc accumulation in grains	[214]
	Iron	Soybean (*G. max* L.)	25–1 M	Seed weight is increased in comparison with normal plants	[215]
		Peanut (*A. hypogaea* L.)	2–1000 ppm	Growth characteristics, root morphology, and productivity were all improved	[75]
		Maize (*Z. mays* L.)	3–5 ppm	The seed germination frequency, the germination time, and the early growth were positively increased	[216]
	Titanium oxide	Barley (*Hordeum vulgare* L.)	500–1000 mg kg^−1^ soil	Nanoparticles are found to enhance plant growth by increasing germination	[217]
		Wheat (*T. vulgare* L.)	5–40 ppm	Plant performance is not affected significantly	[218]
	Titanium oxide activated carbon	Tomato (*S. lycopersicum* L.) and mungbean (*Vigna radiates* L.)	0–500 ppm	It is possible to enhance tomato and mungbean seed germination rates using the appropriate concentration of nanoparticles	[219]
	Silver	Soybean [*G. max* (L.) Mell.]	31.2–62.5 mg/kg soil	Affects seed development and nitrogen fixation negatively	[220]
	Silica	Fababean (*V. faba* L.)	1–3 mM	The seeds germinated more rapidly and grew longer, produced more biomass, improved seed quality, and produced more nitrogen, phosphorous, potassium, calcium and sodium	[221]
Nanopesticide	Silver	Soybean [*G. max* (L.) Mell.]	70 ppm	Pesticidal silver nanoparticle-based treatment for *Callosobruchus chinensis* provided the least amount of seed damage, a minimum seed weight loss, and total mortality	[222]
	Alumina	Wheat (*T. vulgare* L.)	62.5–500 ppm	The nanostructured alumina has been shown to exhibit potential insecticidal effects against *S. oryzae*, which may help improve wheat kernels	[223]
	Silica	Maize (*Z. mays* L.)	0.0031–10 g/kg	For better storage of maze seeds, Nanosilica showed insecticidal effects against *S. oryzae, Rhizoper thadominica, Tribolium castaneum* and *O. philussurinamenisis*	[224]
		French bean *(P. vulgaris)*	400 mg/kg	Insects (*O. surinamensis, Stegobium paniceum, T. confusum)* are controlled during seed storage with Nanosilica as an effective insecticide	[225]
		Peanut (*A. hypogaea* L.)	0.67 mg/kg and 1.7 mg/kg	In groundnut storage, silica nanoparticles showed strong defensive properties against *Caryedon serratus*. Mortality rates increased with nanoparticle exposure time	[226]
	Aluminum oxide	Wheat (*T. vulgare* L.)	8000 mg/kg	A significant decrease in the number of *S. oryzae* offspring was a dose-dependent phenomenon	[227]
	Zinc Oxide	Mung bean (*V. radiata*)	200 ppm	Nanozinc oxides showed the highest mortality (100% of *C. maculatus*) and the lowest egg production in green gram storage	[228]

### Nanofertilizer for Seed Germination

The nanofertilizers are considered to be promising candidates for the fertilizer industry, and they hold the promise of improving nutrient retention in seed and crop growth [62]. Seeds contain ample food reserves that support germination and seedling growth. For seed germination and seedling establishment, the starchy endosperm is the major tissue that accumulates seed reserve food material. The results of several studies have demonstrated that nanofertilizers can assist seeds in germinating in a way that conventional fertilizers cannot. In light of these studies, it is clear that nanofertilizers penetrate seed coats due to their nanosize and increase water absorption by upregulating aquaporin genes, thereby enhancing seed germination and reducing adverse effects of salinity, drought, and heavy metal stresses. Most importantly, many studies have demonstrated that nanofertilizer is not directly applied to the seeds with soil, pre-sowing treatment is beneficial with nanofertilizer, so seeds are in direct contact with nanoparticles in aqueous medium, and seed germination gets induced. Here, nanofertilizer acts as a nanocatalyst for enhancing starch degradation enzyme activity, acting as a mild stress inducer or ROS generator, and creating nanopores in the seed coat. The nanofertilizers also bring positive effect in the microbial communities around seeds, therefore, indirectly contribute to the seed germination [63]. Compared with conventional fertilizers, nanofertilizers are a more effective means of absorbing and utilizing nutrients. This is due to a considerable reduction in leaching and volatilization losses [63]. Unlike chemical fertilizers, the nanofertilizers diffuse freely through soil structures. As nanofertilizers have considerably smaller losses, they can be applied in smaller quantities. This is in contrast to synthetic fertilizers, which require greater quantities in order to compensate for their significant loss through leaching and emission [63]. Nanofertilizers with polymeric coats avoid premature contact with soil and water. Thus, loss becomes negligible, and nutrient contents of nanofertilizers become available when plants are in a position to internalize the released nutrients [64].

Nanofertilizers containing nitrogen, phosphorous, and potassium have been found to improve plant development and crop production [65]. Nitrogen is the principal mineral element required in the biosynthesis of amino acids, proteins, nucleic acids, enzymes, hormones, vitamins, secondary metabolites, etc. Nitrogen plays a key role in photosynthesis as it is the main constituent of chlorophyll. For plant growth, phosphorous is the second most abundant nutrient. It plays a critical role in the synthesis of nucleic acids, phospholipids, and phosphor-proteins. Furthermore, it is the main component of the metabolic energy source adenosine triphosphate. Among the primary nutrients, potassium is the third-most important and controls a number of metabolic processes, such as transport, opening and closing of stomata, controlling cytoplasmic pH, and activating more than 60 enzymes. Potassium is known to enhance the defense mechanism of a plant. Synthetic fertilizers release their nutrients in 4–10 days, whereas nanofertilizers release them in 40–50 days [66]. Furthermore, nanofertilizer increases the tolerance of seeds to both biotic and abiotic stresses by triggering many molecular mechanisms [67]. Badran and Savin [68] successfully studied the seed germination and early stages of bitter almond seedlings' growth under saline conditions using nanofertilizer (nanourea modified with hydroxyapatite nanoparticles). A copper oxide-based tenorite nanofertilizer was effectively developed by Esper Neto et al. [69], for the growth of corn seedlings. Using the seed priming technique (pre-soaking of seeds in colloidal solution of nanoparticles), Abdel-Aziz et al. [70], investigated the effects of engineered nanomaterials alone or in combination with nitrogen, phosphorous, and potassium on the growth and productivity of French beans. Kumar Das et al. [71] developed a sustainable design for rice production using nitrogen, phosphorous, and potassium fertilizer equivalent nano-pyrite seed dressing. Using zerovalent iron nanofertilizer, Titir Guha et al. [72] improved the germination of aromatic rice (*Oryza sativa* cv. *Gobindabhog* L.). Kubavat et al. [73] synthesized a chitosan-based sustained release nanofertilizer formulation to improve the biomass production of *Zea mays*. In the study by Abdel-Aziz et al. [70] engineered carbon nanotubes nitrogen, phosphorous, potassium and chitosan nanoparticles nitrogen, phosphorous, potassium fertilizer was effectively tested on the growth of French beans (*Phaseolus vulgaris*)*.* A novel nanofertilizer synthesized by Yusefi-Tanha et al. [74] has been evaluated for its influence on soybean seed yield (Glycine max cv. Kowsar). A study by Rui et al. [75] suggested using iron oxide nanoparticles as a potential iron fertilizer for peanuts (*Arachis hypogea*). These are a few recent examples of nanofertilizers that were used to promote seed germination and seedling growth. A very limited amount of research is being done in the area of nanofertilizers for seed germination and development. More research is needed in this area.

### Nanopesticides for Seed Protection

Using nanotechnology to enhance pesticide delivery could improve pesticide utilization and reduce runoff into the environment, thus reducing environmental pollution and negative impacts caused by pesticides [76]. On plant surfaces, nanopesticide formulations can improve droplet adhesion, increasing the dispersion and bioactivity of active ingredients. Therefore, nanopesticides are more effective at controlling crop pests than conventional pesticides. Nanopesticides not only improve pesticide dispersion but also enhance their bioavailability by accelerating the delivery of their beneficial ingredients. Consequently, nanopesticides are widely used to reduce the shortcomings of conventional pesticides, such as their low efficacy and large doses. In contrast to conventional pesticide formulations, nanopesticides release the active ingredient slowly at a predetermined rate to achieve their desired efficacy and longevity. By encapsulating pesticides in nanopesticides, the active ingredients of pesticides are protected from premature degradation and direct release to mankind. Unlike conventional pesticides, nanopesticides have a large surface area, which increases their ability to interact with target pests at a lower concentration [77]. Thus, the application of nanopesticides can be a sustainable option for increasing the crop productivity. Nanopesticides are pesticides formulated in the nanoscale for agricultural applications, whether pesticides are fixed on a nanomaterials, encapsulated in a matrix or embedded in enzyme- or stimuli-triggered nanocarriers [78]. The term nanopesticide refers to any nanochemical that kills pests, including weeds, insects, bacteria, and fungi [79]. Nanomaterials like silver, gold, iron oxide, titanium oxide, copper oxide, and zinc oxide are antimicrobial and anti-insecticidal, so they are ideal for use as nanopesticides. Despite their nanostructure and small size, nanomaterials can penetrate cell membranes and attach to cell organelles, causing abnormal oxygen species to form and causing an alteration to normal cell functioning. A normal level of ROS is necessary for important physiological processes, such as cell signaling, gene expression, and protein redox regulation; however, high levels of ROS cause anomalies and interfere with normal functioning, which results in cell death. Another molecular mechanism of nanopesticides for seed protection involves inhibiting cell wall synthesis, depolarising the cell membrane, inhibiting protein synthesis, inhibiting amino acid synthesis, and inhibiting metabolic pathways in pests and microorganisms [80].

Nanopesticide-mediated ROS not only kill seed pathogens, but also enhance seed and plant defense by activating antimicrobial peptides and secondary metabolites in plants raised from nanotreated seeds. Plant secondary metabolites play an important role in their defense, communication, and adaptation, but their secondary metabolism in response to nanoparticles is not completely understood. Several studies [81, 82] demonstrate that nanoparticles trigger ROS production significantly across plant species, which result in the synthesis of antimicrobial secondary metabolites; however, their exact molecular mechanisms are unknown. Although it is clear that ROS are involved in transcriptional regulation of antimicrobial secondary metabolites, there is also a link between ROS and secondary signaling messengers [81]. Thus, ROS generated by nanoparticle interactions may interfere with plant secondary metabolism and cause plants to produce antimicrobial secondary metabolites to defend themselves from pathogens. A nanofungicide made of iron nanorods was successfully used to inhibit the growth and fabricated zinc oxide nanoparticles as a tool for controlling soybean seed-borne phytopathogenic fungi was studied by Lakshmeesha et al. (2021) [83]. Almaary et al. [84] explored the application of seed-borne *Penicillium duclauxii* to the synthesis of silver nanoparticles. The comparative pot studies of chitosan and chitosan-metal nanocomposites as nanofungicides were conducted by Kaur et al. [85] against *fusarium wilt* of chickpea (*Cicer arietinum* L.) using triazolyl dithiocarbamate. A potent antifungal nanosilver agent has been developed by Sharma et al. [86] against bakanae disease of rice. The novel study was delivering pesticides to plant parasitic nematodes using tobacco mild green mosaic virus as a nanocarrier was carried out by Chariou et al. [87]. Sankar and Abideen [88] investigated the nanopesticidal effects of silver and lead nanoparticles against the pest *Sitophilus oryzae*. To protect the faba bean (*Vicia faba*) from insects, Thabet et al. [89] investigated silica nanoparticles as potential nanopesticides. A nanoformulation of thiosemicarbazone has been developed by Spadola et al. [90] to control fungus *Aspergillus flavus* infection in grains. The above-mentioned nanopesticides-based studies highlighted their potential for use in seed science and technology. As seen in these reports, a substantial amount of research can be conducted in the near future to develop nanopesticides for seed-borne infection and storage.

## Nanoparticles: Potential Tool for Seed Treatment

The nanoparticles in nanoformulations are the only constant component, and they keep changing with the type of product. Nanoparticles are classified into inorganic and organic nanomaterials according to their chemical composition. Inorganic and organic nanoparticles are used as promising agents for seed priming, coating, and pelleting (Table 2). Listed below are some examples of most effective nanoparticles that can be used for the designing of seed-specific nanoformulations.

**Table 2:** An overview of the nanoparticle systems used in seed priming and coating, their characteristics, and their main effects on different seed species

Type of nanomaterial	Size in nanometers	Concentrations for seed treatment	Seed type	Key findings	References
Zinc oxide	20–30 nm	25–100 ppm	Wheat (*T. aestivum L*.)	Reduce cadmium uptake	[105]
	15–52 nm	5–200 ppm	Rice (*O. sativa L*.)	Improved biofortification	[229]
	35–40 nm	750–1250 mg/kg	Chili (*C. annuum L*.)	High antimicrobial activity	[230]
	40 and 60 nm	1–5000 ppm	Common bean (*P. vulgaris L*.)	Improved biomass	[98]
	21.3 nm	20–60 mg/L	Lupin (*Lupini stermis L*.)	High salinity resistance	[99]
	32 nm	50–500 ppm	Pearl millet (*Pennisetum glaucum L*.)	Antimicrobial resistance	[100]
Iron	50 nm	10–500 ppm	Sorghum (*S. bicolor (L.) Moench*)	Increased water content in leaves	[91]
	19–30 nm	20–160 ppm	Watermelon (*Citrullus lanatus (Thunb*.) Matsum and Nakay varieties)	Increased the activity of plant growth regulator	[231]
	6–20 nm	30 µg/mL	Rice (*O. sativa L*.)	High antimicrobial activity	[93]
	80 nm	25–1000 µg/mL	Wheat (*T. aestivum L*.), types WL711 (low-iron genotype) and IITR26 (high-iron genotype)	Increased harvest yield	[95]
	33.8 ± 3.59 nm	10–160 mg/L	Rice (*O. sativa* L.)	Improved water uptake	[72]
	25–100 nm	50 µg/mL	Rice (*O. sativa* L.)	Improved enzymatic activity	[71]
	20–30 nm	20–40 ppm	Wheat (*T. aestivum* L.) seeds of varieties galaxy-13, Pakistan-13, and NARC-11	It develops abiotic stress resistance in wheat	[103]
Manganese (III) oxide	50 nm	0.1–1 mg/mL	Jalapeño (*C. annuum* L.)	Salinity resistance development	[105]
Copper	25, 40, and 80 nm	1–1000 mg/L	Common bean (*P. vulgaris* L.)	High concentrations showed toxic effects on seed germination	[102]
	15–30 nm	20–40 ppm	Wheat (*T. aestivum* L.) seeds of varieties galaxy-13, Pakistan-13, and NARC-11	Abiotic stress resistance development	[102]
Platinum	3.2 ± 0.8 nm	Concentrated solution at 1.0 mM	Pea (*P. sativum* L.)	Decreased microorganism’s colonization	[198]
Carbon	13–14 nm	70 µg/mL	Wheat (*T. aestivum* L.)	Improved harvest	[232]
Molybdenum	35–50 nm	10 mg/L	Chickpea (*C. arietinum* L.)	Increased antioxidant enzymes and harvest	[233]
Silver	6–26 nm	10 and 20 mg/mL	Rice seeds (*O. sativa L.* cv. KDML 105)	Increased aquaporin gene expression	[119]
	11.6 ± 2.40 nm	31.3 µg/mL	Onion (*A. cepa* L.)	Potentially increased biochemical activity	[120]
	10–35 nm	0–50 mg/L	Wheat seeds (*T. aestivum* L.)	Increased seed and seedlings vigor	[121]
	5–30 nm	10–50 µg/mL	Soybean (*G. max* (L.) Merr.)	Potential antimicrobial activity	[123]
Gold	10–30 nm	5–15 ppm	Maize (*Z. mays* L.)	Improved seed and seedlings vigor	[124]
	93.68 ± 2.06 nm	31.3 µg/mL	Onion (*A. cepa* L.)	Improved seed and seedlings vigor	[66]
Silica	90 nm	300–1200 ppm	Wheat (*T. aestivum* L.)	Reduced cadmium uptake	[234]
	~ 100 nm	2 mg/mL	Pea seeds (*P. sativum* L.)	Improved seed and seedlings vigor	[18]
Chitosan	259.4 ± 4.7 nm	1–100 µg/mL	Wheat (*T. aestivum L*.)	Increased plant growth regulator (auxin)	[235]
	95 ± 2 nm	20 µg/L	Common bean (*P. vulgaris* L.)	Increased ROS levels	[69]
	122 nm	0.05–0.2%	Rice (*O. sativa* L.)	Potential antimicrobial activity	[129]
	560 nm	0.1%	Chickpea (*C. arietinum* L.)	Improved activity of plant growth regulator	[236]
	450 ± 10 nm	0.05–0.0005 mg/mL	Tomato (*S. lycopersicum var*. *cerasiforme*)	Improved harvest yield	[237]
	400 nm	250 mg/kg	Pearl millet (*P. glaucum*)	Improved plant growth regulators	[132]
	374.3 ± 8.2 nm	0.01–0.16% w/v	Maize seeds (*Z. mays* L.)	Improved seed and seedlings vigor	[133]
	387.7 ± 4 nm	0.01–0.16% w/v	Maize seeds (*Z. mays* L.)	Development of biotic resistance and improved harvest yield	[101]
Lignin	200–250 nm	0.5, 1, and 1.5 mg/mL	Arugula (*Erucavisicaria* L.) Cav. subsp. sativa), tomato (*S. lycopersicum* L. cv. Ciliegino), and chickpea (*C. arietinum* L.)	Improved seed and seedlings vigor	[137]

### Inorganic Nanoparticles

#### Iron Oxide Nanoparticles

Iron oxide nanoparticles at low concentrations promoted the growth and development of seeds. A study by Maswada et al. [91] demonstrated the potential of nanoiron oxide to improve sorghum (*Sorghum bicolor*) germination and seedling growth using soaking and priming of seed under salinity conditions. Kasote et al. [92] used onion extract to synthesize iron oxide nanoparticles. It was shown that non-toxic iron oxide nanoparticles could be applied sustainably to watermelon seeds to increase anti-inflammatory properties and enhance defenses. Using aqua dispersed nanoparticles of ferrous sulfide, Ahuja et al. [93] assessed the phytopathological effects against the rice born fungus *Fusarium verticillioides.* Nanoiron treated rice seed showed significant seed germination and inhibition of fungus *F. verticillioides* [93, 94]. Sundaria et al. [95] suggested that biofortifying wheat with iron through seed priming could address anemia caused by iron deficiency. The given treatment showed a significant increase in grain iron content and higher accumulation. Guha et al. [72] reported the use of nanopriming using zerovalent iron to increase germination and growth in aromatic rice cultivars. Das et al. [71] proposed a seed dressing approach for rice, and their experiments revealed that the nano-pyrite seed dressing triggers nitrogen, phosphorous, potassium equivalent rice production without compromising yield. Thus, the above-mentioned iron oxide nanoparticles could be used as a platform for further asset delivery system development. Iron oxide nanoparticles can significantly reduce the presence of iron in the cotyledon, which inhibits the uptake and translocation of nanoparticles. Furthermore, these iron oxides are externally aggregated on seeds, making them ideal for seed priming and pest control applications.

#### Zinc Oxide Nanoparticles

Seeds require zinc for many physiological and biochemical processes. Many studies show that seed primed with zinc oxide nanoparticles has a higher zinc content, which contributes to a higher yield and higher growth rate***.*** According to Rizwan et al. [96], zinc oxide nanoparticles positively affected wheat growth and decreased cadmium accumulation in wheat. Zinc nanoparticles enhanced zinc concentrations in roots, shoots, and grains. Overall, nanoparticles significantly increase wheat biomass, and nutrient retention, and decrease cadmium toxicity. In a study by Itroutwar et al. [97], biogenic nanozinc was synthesized for rice seeds using brown seaweed extract *Turbinaria ornata*as a priming agent, resulting in increased rice seed quality and crop yield as a result. Zinc nanoparticles were used by Savassa et al. [98] to enhance seed nutrition. This study evaluated the effects of different concentrations and sizes of zinc nanoparticles on bean (*P. vulgaris*) seed germination. Biotransformation of zinc oxide nanoparticles was detected using X-ray absorption spectroscopy. It was found that most of the zinc absorbed by seed coat was trapped there, while a small fraction entered cotyledons. The results demonstrate potential for using zinc nanoparticles as an agrochemical due to their properties, particularly the slow zinc release and lower toxicity than zinc sulfate. According to Latef et al. [99], nanozinc effectively prevented seed germination loss of *Lupinus termis* seeds grown under salinity stress. As a result, zinc nanoparticles may boost the growth and yield of plants growing in salinized soils. The mechanisms by which zinc oxide nanoparticles alleviate the adverse effects of salinity stress in seeds require further study. The use of biofabricated nanozinc as a potent seed priming agent for growth promotion and mildew control in pearl millet was recently reported by Nandhini et al. [100]. Chudhary et al. [101] fabricated the zinc–chitosan nanoparticles and assessed them via seed priming and foliar application in maize. Zinc–chitosan nanoparticles have been shown to act as antifungals and promote seedling growth. Above all, the studies showed that nanozinc could be utilized in agriculture, but a thorough understanding of their interactions with seeds is needed.

#### Copper Oxide Nanoparticles

A number of enzymes are activated by copper, which contributes to RNA synthesis and improves photosystems’ performance. A range of copper oxide nanoparticle sizes and concentrations influence *P. vulgaris* seedling germination and growth. According to Duran et al. [102], it was found that most copper was in its pristine form through X-ray absorption spectroscopy. Seed germination was not affected by copper nanoparticles, but seedling weight gain was promoted at low concentrations but inhibited at high concentrations. Biosynthesized copper oxide nanoparticles markedly induced and promoted antioxidant enzyme activities. The potential role of nanocopper in wheat yield was studied by Yasmeen et al. [103]. Size- and dose-dependent toxic activity was observed for the biosynthesized copper oxide nanoparticles against two wheat grain damaging insects, *Sitophilus granarius* and *Rhyzopertha dominica*. Findings suggested that copper oxide nanoparticles should be used at lower concentrations in agricultural fields as insecticides that will not inhibit wheat growth. A study by Wang et al. [104] examined the concentration-dependent effects of copper oxide nanoparticles on the germination, growth, and physiological responses of *Brassica pekinensis* L. In light of these results, nanocopper can serve as a powerful insecticide to facilitate the storage of a wide range of seeds and, at lower concentrations, it can significantly improve the seed germination rate.

#### Manganese Oxide Nanoparticles

It was known that manganese helps enhance the seed germination rate. It has been found that nanoscale manganese is less phytotoxic and more effective at minimizing abiotic stresses compared to conventional bulk or ionic manganese compounds. There is little information available regarding the physiological and toxicological effects of manganese nanoparticles on agricultural crops. Manganese oxide nanoparticles were studied as a nanopriming agent by Ye et al. [105] to alleviate salinity stress in the *Capsicum annuum* during germination. According to the study, the surface charge plays an important role in the behavior of nanomanganese oxide. Manganese oxide nanoparticles have been used as seed priming agents to improve chlorophyll and antioxidant profiles in watermelon seedlings by Kasote et al. [106]. Similarly, bio-engineered magnesium oxide nanoparticles [107] were shown to enhance green gram seedling strength. However, more research is needed to determine the exact effect of seed priming with manganese on agricultural output, including its role in enhancing abiotic and biotic tolerance.

#### Cobalt Nanoparticles

The micronutrient cobalt is another essential one. Cobalt concentrations influence the response of seeds. It promotes plant growth in low concentrations but can cause phytotoxicity at higher concentrations. Hong et al. [108] investigated the effect of nanoscale zerovalent cobalt on soybean growth. Soybean growth and development were positively influenced by zerovalent cobalt at nanoscales. Krishnamoorthy et al. [109] investigated hexa-amino-cyclotriphosphazene and cobalt nanoparticles incorporating polyvinylpyrrolidone seed coatings for improving cowpea seed germination. Cobalt-coated seeds showed higher imbibition rates, which could help reduce drought stress. Nanocobalt-based seed coating given to a seedling could increase germination rates and enhance stand establishment.

#### Carbon Nanoparticles

Using multi-walled carbon nanotubes as a nanopriming agent, Joshi et al. [110] developed a new nanopriming agent. The use of multi-walled carbon nanotubes in wheat significantly increases seed yield. The effects of carbon-based nanomaterials on seed germination under salt stress were investigated by Pandey et al. [111], and nanocarbon has been described as promising seed germination and plant growth product. Carbon nanomaterials are promising seed germination promoters and plant growth regulators. Baz et al. [112] reported that carbon nanoparticles might enhance seed germination and post-germination growth of lettuce under salinity stress. The study showed that soluble nanoparticles improved lettuce seed germination under salt stress, which provides fundamental evidence about the potential of nanoparticles in agricultural applications to improve crop yield and quality.

#### Graphene Oxide Nanoparticle

A graphene oxide is a unique material that consists of a single monomolecular layer of graphite with epoxide, carbonyl, carboxyl, and hydroxyl groups containing oxygen functionality. Recently, graphene oxide nanoparticles have been investigated as seed stimulators in agriculture for improving seed germination, seedling growth, and further development. According to Yin et al. [113], graphene oxide significantly impacted the germination and growth of seeds, as well as the uptake of cadmium in solution cultures. As a result of this study, graphene oxide can inhibit the adverse effects of cadmium on seed germination, seedling growth, and uptake of cadmium in solution, and positively stimulates the seed germination. In another study, Kim et al. [114] demonstrate that silver–graphene oxide nanoparticles can significantly affect the early growth of seeds depending on the species. In rice seeds, Li et al. (2020) demonstrated the significant effects of graphene oxide nanosheets on rice seed growth. Based on these findings, graphene oxide nanosheets were found to inhibit the adverse effects of cadmium on rice seed germination and to reduce cadmium uptake and accumulation in rice seedling roots and shoots, helping to determine cadmium’s fate and ecotoxicity [115]. As a result, graphene oxide nanoparticles can be utilized as a potential seed stimulant.

#### Silicon Nanoparticles

Silicon decreased malondialdehyde levels in radicles under stress, indicating a decreased lipid peroxidation rate. Exogenous silicon increased antioxidant defense in bud seedlings, improving seed germination and alleviating oxidative stress. Cadena et al. [116] demonstrated the enhancement of cinnamon essential oil activity using nanoparticle encapsulation to control seed pathogens. To combat seed-borne diseases, cinnamaldehyde-mesoporous silica nanoparticles were incorporated into a sodium alginate seed coating. Hussen et al. (2019) [117] determined the effects of nanosilica on wheat growth, yield, and cadmium accumulation. Rahimi et al. (2021) [118] investigated the potential role of silicon nanoparticles in affecting seed germination and vigor of calendula (*Calendula officinalis* L) under drought stress induced by polyethylene glycol.

#### Silver Nanoparticles

**Fig. 3:**
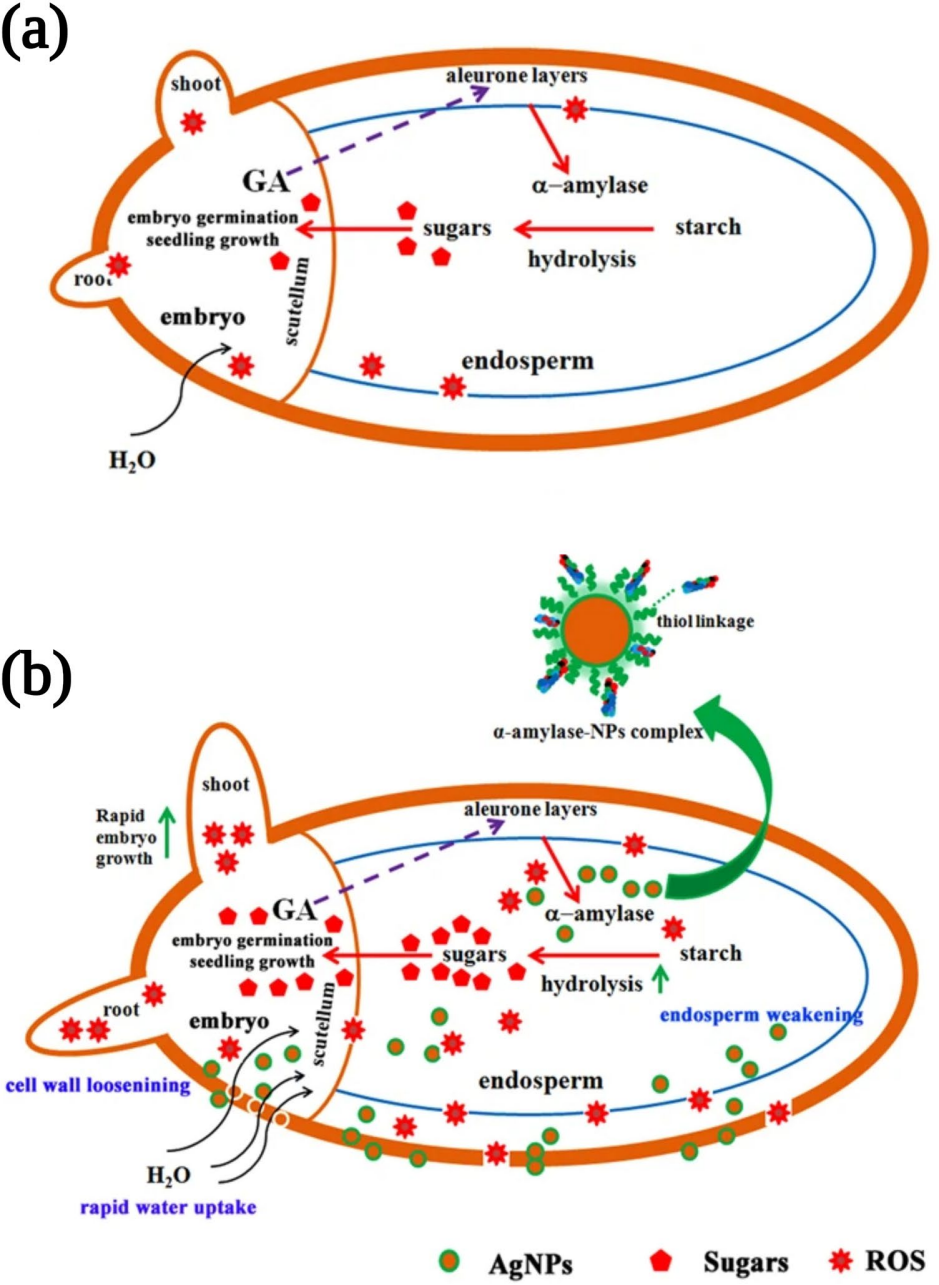
Phytosynthesized silver nanoparticles enhance aged rice seeds’ germination and starch metabolism. **a** Seeds without silver nanoparticle priming treatment have lower metabolic activity because of slow water uptake, and starch is hydrolyzed slowly; as a result, sugar levels are low in the initial stage of imbibition, resulting in slow seed germination and growth. **b** Silver nanoparticle seed priming enhances seed germination. This is a reprinted image of Ref. [119] with permission

A wide range of nanomaterials are used in agriculture research, including silver nanoparticles. According to Mahakhham et al. (2017) [119], the mechanism of silver nanoparticles promoting seed germination has been hypothesized as (i) functioning as a nanocatalyst for enhancing starch degradation enzyme activity, (ii) acting as a mild stress inducer or ROS generator, and (iii) creating nanopores in the seed coat. Silver nanoparticles can be used as a nanopriming agent for enhancing seed germination and starch metabolism of rice-aged seeds. As depicted in Fig. 3, the nanopriming of silver could cause an increase in the activity of amylase, leading to a higher content of soluble sugars that would support seedling growth. As a result of nanopriming treatment, amylase activity increased, resulting in a higher concentration of soluble sugars. As sugar concentrations increase in the cells, the osmotic potential and water potential decrease. As a result, the difference (gradient) between the water potential outside and inside the tissues increases, allowing water to move into the seeds through osmosis. Due to the increased soluble sugar content and amylase activity in nanoprimed seeds, the increase in water uptake may also be due to the change in internal osmotic potential caused by soluble sugars (solutes). In Mahakhham et al. (2017) [119] study, ROS, including hydroxyl radicals, were shown to be important for cell wall loosening, testa, and endosperm weakening, which is necessary for radicle protrusion.

The nanopriming treatment produced higher ROS levels in germinating seeds than the unprime control or other priming treatments, suggesting both ROS and aquaporins contribute to seed germination. There was evidence that nanosilver can internalize seed coats and support water uptake inside seeds, thus promoting seed germination and starch metabolism. Several mechanisms have been proposed for nanopriming-induced seed germination, including the creation of nanopores that enhance water absorption, rejuvenation of ROS/antioxidant systems in seeds, and the generation of hydroxyl radicals that loosen the cell wall. As seed nanopriming maintains ROS levels within the oxidative window that promotes seed germination, seed nanopriming increases seed germination. Nanoparticles can reduce the level of ROS in seeds under stress conditions, as they increase the activity of enzymes like superoxidase dismutase, catalases, and guaiacol-peroxidase. This reduces seed cell damage. According to the study of Mahakhham et al. (2017), silver nanoparticles penetrated seed coats and created small pores, which resulted in increased water uptake and increased expression of aquaporin genes involved in water uptake. Acharya et al. (2019) [120] synthesized nanosilver from onion extract, which was internalized by onion seeds. Several greenhouse and field studies have demonstrated that seeds germination, growth, and yield are significantly enhanced. The study by Kannaujia et al. [121] demonstrated the potential role of biogenic nanosilver on wheat growth as a growth promoter without any toxic effect typically associated with chemically synthesized nanosilver. Using agro-industrial by-products, Acharya et al. (2020) [122] synthesized silver nanoparticles and used them as nanopriming agents for diploid and triploid watermelon seeds. Seed treatment with nanosilver has been demonstrated to improve seed germination, growth, and fruit quality. Spagnolettia et al. (2019) [123] obtained stable silver nanoparticles from the exudate of the soil fungus *Macrophomina phaseolina* using a low-cost, green synthesis process. The effect of silver nanoparticle dosage on soybean seed germination was also studied to test its potential applicability as a seed protection agent.

#### Gold Nanoparticles

The properties of gold nanoparticles make them attractive candidates for seed priming applications since they have a small size, good biocompatibility, low toxicity, easy surface chemistry, and easy surface modification. According to Mahakhham et al. (2016) [124], a nanogold solution was used to elicit seedling growth and germination in aged maize seeds. A study showed that the nanopriming approach minimized gold translocation from seeds into plant vegetative organs. Gopinath et al. (2014) [125] studied the effects of gold nanoparticles produced from fruit extract of *Terminalia arjuna* on *Gloriosa superba* seed germination. It was found that nanogold significantly affected seed germination and vegetative growth of *Gloriosa superba*. A study by et al. (2017) [126] examined the effect of green synthesized gold nanoparticles on rice germination and root growth. Overall, the results indicated that gold nanoparticles synthesized by *Tiliacora triandra* can enhance seed vigor and are biocompatible. *Brassica juncea* growth and seed yield are enhanced by gold nanoparticles, as demonstrated by Arora et al. (2012) [127].

### Organic Nanoparticles

Nanoparticles made from natural or synthetic organic molecules are called organic nanoparticles. An organic nanoparticle can be made from a polysaccharide, lipids, or proteins that are biodegradable, biocompatible, and able to react to various environmental stimuli like pH, temperature, etc. [128]. Organic nanoparticles can be effective carriers of seed health-promoting compounds when applied as seed coatings or seed dressings material. A wide selection of chemicals can be loaded into these nanoparticles, including fungicides, essential oils, plant growth regulators, and fertilizers.

#### Chitosan Nanoparticles

Nanoparticles made of chitosan are biodegradable, more stable, less toxic, and biocompatible. Li et al. (2018) [129] examined the effect and mechanism of chitosan nanoparticles on wheat germination and seedling growth. As a result of the higher adsorption of chitosan nanoparticles on the surface of wheat seeds at low concentrations, chitosan nanoparticles provide beneficial effects to the growth of wheat seeds. A chitosan guar nanoparticle was prepared by Sathiyabama et al. (2020) [129] with high antimicrobial activity as a bioprotectant against rice phytopathogens. According to this study, chitosan guar nanoparticles can be used as an antimicrobial agent to combat rice blast and blight disease. Nanochitosan loaded with nitrogen, phosphorous, and potassium is tested as a fertilizer for french beans by Azizi et al. (2019) [69]. According to the obtained results, nanochitosan loaded with nitrogen, phosphorous, and potassium might be used to improve seed germination. A study by Divya et al. [130] optimized the synthesis of chitosan nanoparticles and investigated their potential use as a germination elicitor for rice seeds. Rice seeds treated with chitosan nanoparticles remained effective when stored at room temperature for seven months. Using nanoalginate–chitosan and nanochitosan–tripolyphosphate containing gibberellic acid, Anderson do Espirito Santo Pereira et al. (2019) [131] designed a seed treatment that improved the growth and productivity of *Solanum lycopersicum* under field conditions. Using chitosan nanoparticles, Siddaiah et al. [132] investigated the effectiveness of the nanoparticles against downy mildew in pearl millet. A study showed that chitosan nanoparticles increased pearl millet germination and seedling vigor after seed treatment with chitosan nanoparticles. The effect of copper–chitosan nanoparticles on physiological and biochemical changes during maize seedling growth was investigated by Saharan et al. [133]. According to studies, copper–chitosan nanoparticles promote seedling growth through better mobilization of reserved food, such as starch, through increased levels of α-amylase.

#### Cellulose Nanofibers

The cellulose nanoparticles are easy to process, cost-effective, biodegradable, and have good solubility in many organic solvents. Biodegradable cellulose biopolymer-based nanofiber seed coatings were used by Xu et al. [134] to enhance agrochemical delivery and seedling development. The greenhouse studies found that nano-enabled seed coatings effectively deliver agrochemicals at the right place while consuming a minimum number of agrochemicals. Zhang et al. [135] developed cellulose anionic hydrogels based on cellulose nanofibers for significant seed germination and seedling growth. The present study provided an easy and effective method for fabricating cellulose anionic hydrogel and evaluated its application in agriculture.

#### Lignin Nanoparticles

Lignin nanoparticles have excellent antibacterial and antioxidative properties due to their surface chemistry and shape. Kacsó et al. [136] used zein and lignin-based nanoparticles to treat soybean seeds. A seed treatment containing azoxystrobin-loaded lignin nanoparticles provided almost complete antifungal protection for soybean against fungus *Rhizoctonia solani*. The results indicate that nanozein and lignin are safe and effective delivery systems for active compounds in seed treatments. Falsini et al. [137] synthesized lignin nanocapsules, which were used as potential vectors for the delivery of bioactive compounds to tomato and miller seeds; it was found that lignin nanocapsules enhanced seed growth and development of tomato and miller seeds. It is necessary to conduct further studies to determine the precise mechanisms responsible for the differential effects of nanolignin on seeds.

## Possible Next-generation Nanoscale Architectures for Future Seed Treatment Formulations

**Fig. 4:**
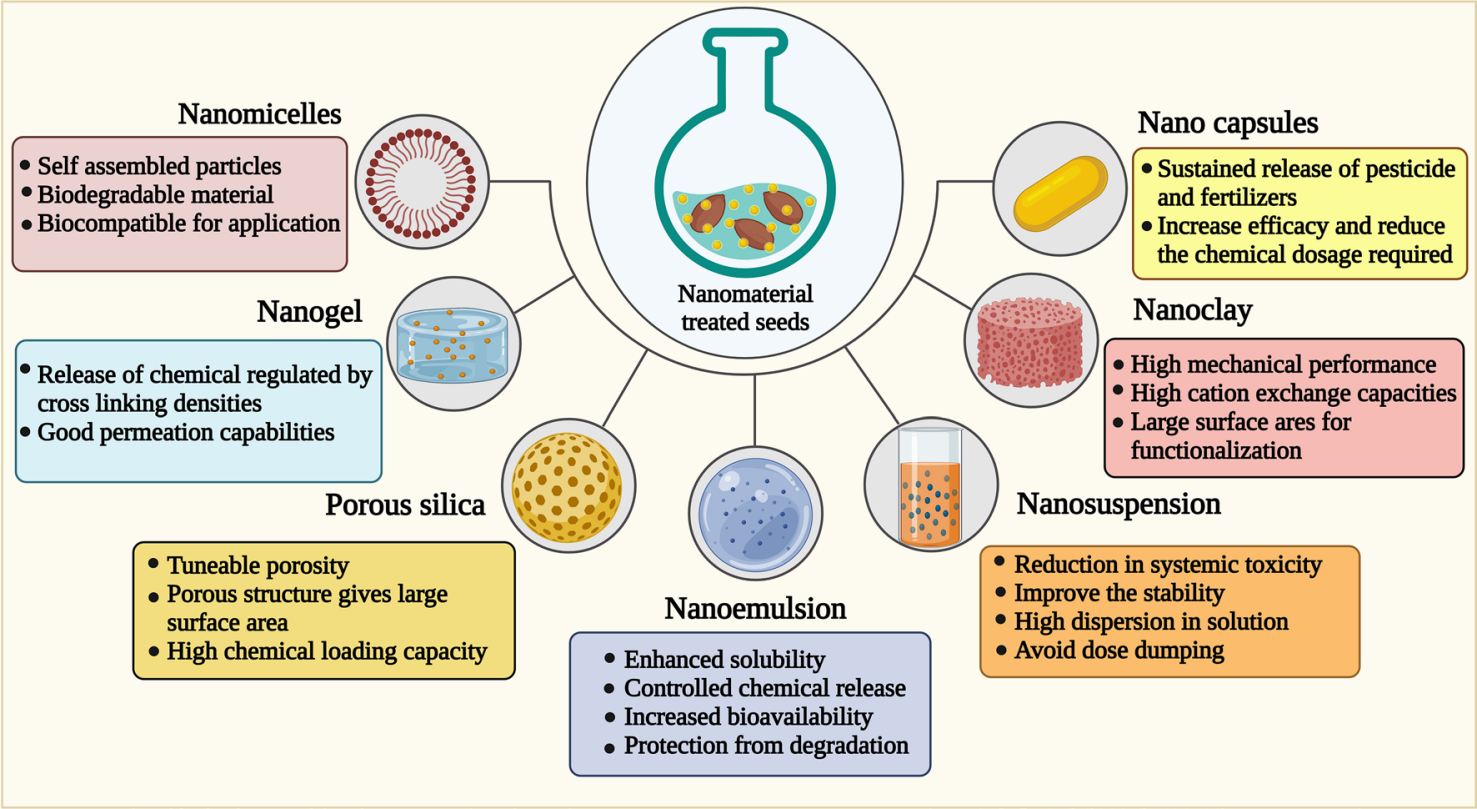
Next-generation nanoagrochemicals for enhancing seed germination (nanoagrochemicals with a wide range of morphologies and structures such as nanomicelles, nanogels, porous silica nanoemulsion, nanosuspension, nanoclay providing controlling stability, solubility, bioavailability, and controlled release of agrochemicals for enhanced seed germinations)

Nanoscale systems of the next generation can control stability, solubility, and bioavailability and provide controlled release of bioactive for seed treatment [138]. Liquid and solid nanoscale systems are the two main types of designing next-generation nanoscale formulations. Using these systems, as depicted in Fig. 4, next-generation seed treatment formulations can be developed for highly effective seed treatments.

### Polymeric Nanocapsules and Nanospheres

The polymeric nanocapsule and nanospheres can be fabricated from preformed polymers or by polymerizing monomers [139]. The nanocapsule and spheres consist of a vesicular or reservoir-type structure with an inner cavity surrounded by a polymer coating or membrane. It may be possible to prepare seed treatments with pesticide/fertilizer-loaded nanocapsules using several different techniques, including nanoprecipitation, emulsion-solvent diffusion, emulsion-solvent evaporation, layer-by-layer self-assembly, ionic gelation, polyelectrolyte complexation, and melt-dispersion techniques [30]. In a double emulsion technique, Kumara et al. (2014) [140] prepared alginate nanocapsules containing imidacloprid and neonicotinoid insecticide. A successful field study on crop pests was conducted to evaluate the effectiveness of imidacloprid nanoformulation. The hydrophilic carbamate insecticide methomyl has been encapsulated in an elegant way by Chuxiang et al. [141]. Nanoencapsulation of methomyl is necessary to prevent early degradation. Chen et al. [142] developed leaf-adhesive pesticide nanocapsules with pH-responsive release to enhance crop leaf retention and improve utilization efficiency. The dual-functionalized pesticide nanocapsule delivery system with improved spreading behavior and enhanced bioactivity was developed by Cui [143]. In contrast to nanocapsules, nanospheres are homogenous, monolithic systems in which the bioactive element is evenly dispersed throughout the polymer matrix. Aza-loaded polymeric nanospheres have been prepared both as suspensions and as powders by da Costa et al. [144]. Using freeze-drying, the colloidal suspension was transformed into powders that provide the best protection against ultraviolet light-induced degradation of the aza in the neem product. Jiang et al. [145] developed lignin–xylan hybrid nanospheres with enzyme-mediated release properties as pesticide carriers. Pectin nanospheres were prepared by Li et al. [146], and their potential impact on wheat seed germination and growth was studied. Here are a few examples of nanocapsules and nanospheres that are effective in their applications. By experimenting with nanocapsules and nanospheres, it is possible to create future formulations that preserve seeds or improve seed storage.

### Nanomicelles

Nanomicelles are self-assembling colloidal particles formed by amphiphilic block copolymers in water. During the formation of a micellar core surrounded by a hydrophilic corona, hydrophobic interactions are developed that drive self-assembly. Zhang et al. [147] developed polyethylene glycosylated-camptothecin nanomicelles to control pesticide combinations. According to this study, micelles could be effective carriers for pesticide combination control. Adak et al. [148] developed nanosized micellar aggregates from amphiphilic copolymers to make controlled release formulations of imidacloprid using aqueous media self-assemble into micellar aggregates upon contacting water. Dong et al. [149] developed pH-responsive ultrasonic self-assembly spinosad-loaded nanomicelles and studied their antifungal activity against *Fusarium oxysporum.* Nanomicelles have not yet been extensively studied for their potential applications in agrochemical delivery. Researchers have the opportunity to explore this nanoarchitecture to design an effective seed treatment system. Biodegradable and environmentally friendly techniques have proven effective seed treatment strategies.

### Nanogels

“Nanogel” generally refers to a water-swollen network of nanoscale polymers, such as hydrophilic or amphiphilic chains that swell without water dissolving. A large surface area of nanogels facilitates multivalent bio-conjugation, and a strong interior network facilitates the incorporation of biomolecules. The transient antiviral activity of chloroinconazide was enhanced by alginate-based nanogel, and its effect on plant growth was studied by Lv et al. [150]. Lv et al. [150] demonstrated the antiviral activity and growth promotion of small molecule pesticides using nanogel carriers for the first time. The composition of the nanogel can easily be applied to the spray-based delivery of pesticides, representing a novel strategy for preparing new pesticide preparations and using multifunctional pesticides to improve seed germination. Ziaee et al. [151] prepared the myristic acid–chitosan nanogel containing essential oil of *Cuminum cyminum* to manage stored product beetle pests effectively. By using this technique, it may be possible to overcome the limitations of essential oils in managing stored product insect infections. It is still necessary to conduct more experiments to optimize nanogels and clarify their toxicity in various commodities, environmental conditions, and insects. However, nanogel has not been fully explored for seed technology, but it could be used to deliver pesticides as it provides pesticidal effects. Nanogel is suitable for use in the initial stages of seed germination and seedling development. Nanogel can be used to coat the seeds with nutrients and maintain moisture while the seeds germination.

### Nanofibers

Nanofibers are fibers that are nanometers in diameter. Fiber diameters ranged from 200 to 400 nm. The nanofibers can be fabricated from various polymers and therefore have a variety of properties and applications. Nanofibers can be synthesized using natural polymers such as collagen, cellulose, silk fibroin, keratin, gelatine, and polysaccharides such as chitosan and alginate [152]. Several methods are available to create nanofibers, including drawing, electrospinning, self-assembly, template synthesis, and thermally induced phase separation. Nanofibers are most commonly generated through electrospinning due to the simplicity of the setup, the ability to synthesize continuous nanofibers from various polymers, and the ability to control the fibers’ diameter, composition, and orientation [152]. Farias et al. [153] used electrospun polymer nanofibers in seed coatings for crop protection. According to Farias et al., localized pesticide delivery can be achieved by coating seeds with cellulose diacetate nanofibers containing abamectin or fluopyram. It is found that nanofibrous coatings electrospun on soybean seeds do not reduce seed germination regardless of coating thickness or uniformity. The in vitro fungal assay performed with fluopyram-loaded nanofibers consistently inhibited the growth of *Alternaria lineariae* [154]*.* Combining sustained release profiles with moisture stability suggest that nanofibrous seed coatings can act as a unique platform to control nematodes and fungi in seeds. Enhancing agrochemical delivery and seedling development with biodegradable, tunable, biopolymer-based nanofiber seed coatings was developed by Xu et al. [134]. Nanofiber-coated seeds (tomato and lettuce) were studied in greenhouse experiments in the presence and absence of a fungal pathogen (*Fusarium sp*) to determine how they germinate and grow over time [155]. This seed nanocoating approach may increase yields in pathogen-infested soil conditions as a result of the precise delivery of agrichemical at the right place while using a relatively small amount of agrochemical. Greenhouse experiments suggest that such nano-enabled seed coating approaches may be useful in pathogen-infested soil conditions [156]. The environment friendly effective seed coat was developed by Krishnamoorthy et al. [157] using electrospun polyvinyl pyrrolidone incorporated with urea and cobalt nanoparticles for use as seed coatings on cowpeas. This new nanofiber seed coating method offers precision in agrochemical delivery and significantly improves germination and seedling biomass for model seeds over conventional film coating methods, due to its unique nanofiber structure and controlled release mechanism.

### Porous Silica Nanoparticles

Agrochemicals can be transported or encapsulated in porous silica nanoparticles because of their biocompatibility, high load capacity and tuneable porosity. Since these nanoporous particles are used in various applications, they can be a viable option for designing nanoformulations for developing seed treatment. Porous silica nanoparticles allow for sustained release of pesticides and fertilizers, intended to increase efficacy and reduce doses required to achieve desired seed effects. Research is underway to establish the potential application of porous silica for effective seed treatments. Sun et al. [158] investigated mesoporous silica nanoparticles ability to enhance wheat and lupin seedling growth and photosynthesis. A dramatic increase in growth was observed in wheat and lupine exposed to mesoporous silica nanoparticles. Furthermore, mesoporous silica nanoparticles localized to chloroplasts in leaves, while photosynthetic activity was markedly increased. The growth and physiological responses of maize to porous silica nanoparticles in soil were studied by Rangaraj et al. [159]. Using nanoscale silica in maize is more effective than bulk silica, thus enabling sustainable agriculture of maize crops as an alternative source of silica fertilizer. Using mesoporous silica nanoparticles, Sattary et al. [160] tested the potential antifungal properties of lemongrass and clove oil against wheat’s take-all disease. In this study, essential oils-mesoporous silica nanoparticles were a safe product to control take-all diseases in wheat crops. Cadena et al. [116] reported improving cinnamon essential oil activity by encapsulating it in mesoporous silica nanoparticles for controlling seed pathogens. Study findings showed that mesoporous silica nanoparticles could be encapsulated to enhance the antimicrobial activity of plant products, thus allowing for the use of volatile biocides, such as essential oils, at very low concentrations to treat and prevent microbial diseases in crops. Study findings showed that mesoporous silica nanoparticles could be encapsulated to enhance the antimicrobial activity of plant products, thus allowing for the use of volatile biocides, such as essential oils, at very low concentrations to treat and prevent microbial diseases in crops. To promote rice seedling growth by regulating amino acid metabolism, Zhao et al. [161] developed mesoporous silica nanoparticles containing fungicides. By regulating amino acid metabolic pathways, fungicide-loaded mesoporous silica nanoparticles protect plants from the negative effects of fungicides [162]. In all of these studies, porous silica nanoparticles have demonstrated that they could potentially be used as novel delivery systems for active ingredients such as pesticides or fertilizers. There is huge scope to explore this material for seed-protecting treatments since very few studies have been done in this field. This material can be a next-generation candidate for developing seed treatment nanoformulations.

### Nanoemulsions

Nanoemulsions are emulsions comprised of droplets with a size on the nanometer scale. They are kinetically stable but are not thermodynamically unstable. Thus, nanoemulsions are metastable systems whose stability depends on the preparation method. Either high-energy or low-energy emulsification methods can prepare nanoemulsions. High-energy methods utilize high-shear stirring, high-pressure homogenizers, and ultrasonic generators, whereas low-energy methods take advantage of the stored energy in the system to produce small droplets. Few reports have been published in which nanoemulsions have been applied in seed treatment. The physic mechanical and antifungal properties of neem oil nanoemulsion for soybean seed coating were studied by Silva et al. [163]. A neem oil emulsion inhibited the growth of fungus *A. flavus* and *Penicillium citrinum* [164–166]*.* Soybean seeds coated with this nanoemulsion showed positive results in the germination process. These new materials have the potential to be used as seed coatings because of their fungicidal properties derived from Neem oil nanoemulsions. Acharya et al. [122] developed a turmeric oil nanoemulsion. This nanoemulsion was successfully used to prime the watermelon seeds of two types of diploids (riverside) and three types of triploids (maxima) using agro-industrial by-products. A new nanoemulsion of eucalyptus oil was developed by Adak et al. [167] to treat two major storage insects (*S. oryzae* (L.) and *Tribolium castaneum* (Herbst) of rice. Compared to eucalyptus oil, their nanoemulsions were superior, and they can be recommended as a safe, non-toxic alternative to harmful chemical pesticides. This small number of studies prompted a willingness to study nanoemulsion development more closely, to design future seeds that contain pesticides or fertilizers.

### Nanoclay

The nutrient-rich nature of clay-containing soils and their ability to retain water make them valuable soils. A nanoclay, also known as layered silicates, is a widely used and studied nanoagent to prepare nanocomposites. A few of the advantages of nanoclays are their widespread availability, easy processing ability, high performance, and low cost. Nanoclay is prepared through a mixing technique in which nanoflakes are dispersed in aqueous media under laminar and turbulent flow conditions. Nanoclay helps deliver micronutrients for crop improvement; it is also used to encapsulate pesticides in nanomaterials for controlled release, stabilizing biopesticides with nanomaterials [168]. Nanoclay has been shown to reduce water usage by up to 50%. Nuruzzaman et al. (2021) [169] studied the ability of organically modified montmorillonite nanoclay to deliver imidacloprid. Taking into account the imidacloprid release pattern from the montmorillonite nanoclay, it can be used as a component in a formulation for a slow-release pesticide where the nanoclay will minimize the instantaneous release of total pesticide. Wang et al. [170] improved the dispersion of nanoclay by incorporating biochar and biosilica to reduce the loss of pesticides. Prepared nanoclay, when added to the pesticide, could effectively increase its adhesion, resulting in a decreased loss of pesticide and reduced pollution risk. Nanoclay application for seed treatment has not received much attention from the scientific community. The potential use of nanoclay in seed coating or pesticide delivery in seeds needs to be explored.

### Nanosuspension

Nanosuspension forms from the dispersion of crystalline or amorphous nanoparticles of the active ingredients in a liquid medium. Nanosuspension preparation has been carried out using several methods, including wet milling, high-pressure homogenization, emulsification, and solvent evaporation. The nanosuspension enhances the solubility and bioavailability of nonsoluble compounds. For this reason, nanosuspension is being considered a possible seed treatment agent in the near future [171]. Zhu et al. (2021) [172] developed a simple method to prepare agrochemical nanosuspensions that combined high concentrations, eco-friendly excipients, and an intensified preparation process to enhance potency. This study shows that flash nanoprecipitation significantly increases the biological potency of agrochemical nanosuspension and reduces their dosage, demonstrating a considerable benefit over traditional preparation methods [173]. Corrias et al. [174] evaluated nanosuspention of zoxamide to improve the solubility of zoxamide and reduce the accumulation and its retention of zoxamide in tomato seeds. The results clearly suggest that nanosuspensions may represent a promising alternative to using poorly soluble pesticides in agriculture. Cui et al. (2019) [175] presented an easy-to-use method of constructing pesticide nanosuspensions through wet milling that improved pesticide (abamectin) bioavailability. Using the highly effective nanoformulation will improve pesticide efficacy, reduce pesticide dosage and reduce environmental pollution. As a demonstration of the potential applications of this system, a formulation was made with carbofuran, a poorly soluble crystalline insecticide. It was found that the nanosuspension system was physically and chemically stable after two years. In all the reports, the effectiveness of nanosuspension in the development of agrochemicals has been demonstrated. The liquid nature of nanosuspension makes it useful for seed priming, seed soaking, and seed coating [176]. Nanosuspension should be explored for seed treatment, as it may benefit seed storage and germination.

## Challenges and Risk Assessment of Nanomaterials-based Agrochemicals

**Fig. 5:**
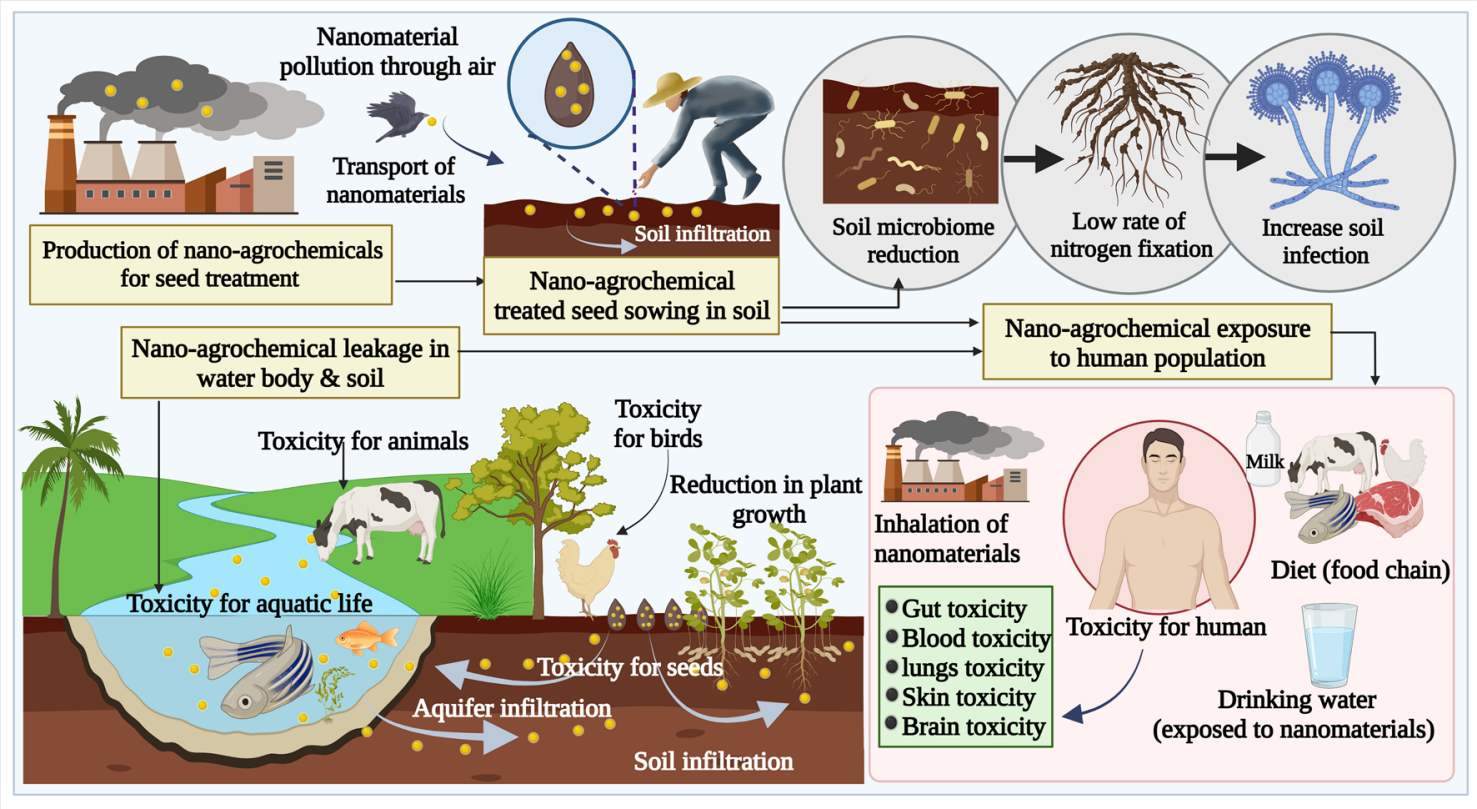
Possible risks associated with nanoagrochemicals-based seed treatments (nanomaterials which made their way into wastewater and soil can contaminate water resource and increases soil pollution. A wide range of soil microbiomes and their nitrogen fixation, mineralization, and plant growth-promoting processes may adversely impact by nanomaterials. A nanomaterial can enter the body of an aquatic organism and livestock can seep into water bodies and enter the food chain and ultimately effecting the human health)

Nanoparticle concentration is key in determining the cytotoxic and genotoxic effects in seed treatment studies, which may vary by agro seed species. Various nanoparticle diameters can enter the nucleus via nuclear pores, indicating that nanoparticles interact with cell components based on their size. Nanoparticles might also disrupt cell cycle checkpoints, enter the cell through mechanical or chemical contact with enzymes that generate reactive oxygen species (ROS), or interfere with cell division mechanisms by binding to proteins and inhibiting protein synthesis. While designing seed treatments using nanomaterials, it is important to consider the properties of nanomaterials, such as size, doses, exposure times, surface chemistry, structures, immune responses, accumulations, and other effects to control the toxicity [177]. Nanomaterials can also enter the environment and soil systems through seed treatment strategies (Fig. 5). Therefore, nanoparticle exposure to seed treatment must be critically assessed and managed in the nanoagricultural field. Research in human and eco-toxicology can provide insight into the complex relationship between the agroenvironment, nanoscale agrochemicals, and human exposure levels [177]. There is an impasse in nanotoxicology regarding how best to assess the risk of nanomaterials-based agrochemicals for environmental monitoring and human health [178]. Toxicology testing can be performed on live (in vivo) organisms, such as microcrustaceans, fishes, mice, other animals and plant models or on cell cultures (in vitro) [179, 180]. Moreover, computational models like quantitative structure–activity relationship provide a lot of potential for understanding the possible toxicity effects of nanomaterial-based agrochemicals on humans and the environment [181, 182]. However, until now, the toxicity of nanomaterial-based agrochemicals is not evaluated according to any standards, making it difficult to compare the results and reach a consensus on their toxicity [78]. There are no standard methods for evaluating nanomaterial-based agrochemicals, as they can present a range of physicochemical properties. So far, most studies have been adapted from standard methods for other substances like drugs, synthetic chemicals, etc. Various assays have been proposed, but there is still no standard protocol. As of now, governments of all nations have not adopted a fixed toxicity regulation strategy for nanoparticle used to assess human and animal health, safety, and ecological impacts.

In the present state of knowledge, the mechanisms of toxicity of nanomaterial-based agrochemicals to plants are largely unknown [183], and little information exists about how nanoagrochemicals might enter seeds, plants and where they may end up in the food chain [184]. However, a few studies have tried to unravel the toxicity effects and mechanisms of different nanoparticle treatments on seeds and plants. A phytotoxicity evaluation method was conducted on seeds of *Allium cepa*, *Z. mays* (maize), *Cucumis sativus* (cucumber) and *Lycopersicum esculentum* (tomato) exposed to nanoparticles [185]. In vitro studies on seeds and seedlings were conducted to determine cytotoxicological effects such as mitotic index, chromosomal aberrations, vagrant chromosomes, sticky chromosomes, disturbed metaphase, breaks, apoptosis, and micronuclei formation. Phytotoxicity endpoints can be evaluated through in vitro and in vivo studies that expose seed systems to different nanoparticle dispersion concentrations to evaluate germination rate, root/shoot length, adsorption, accumulation, and translocation of the nanoparticles [186, 187]. The toxicity of many nanoparticles was assessed using biochemical studies on protein expression analysis, DNA consistency after nanoparticle treatment, and enzyme levels in seeds, seedlings and plants [188]. Seed structure and toxicity effects can be studied with advanced microscopic techniques like electron microscopy. They can reveal nanotoxicity by using focused electron beams rather than visible light. These approaches can better understand the risks associated with nanomaterials and their products before using them directly on the field for seed treatment. Plant model organisms such as *Arabidopsis* are useful for genetic experiments because it has several important characteristics such as a short generation time, small size, and prolific seed production through self-pollination. *Arabidopsis* is useful in toxicity testing of nanomaterials to analyze the mutations, accumulation and translocation, physiological responses, and oxidative stress generation during the interaction of nanomaterials [189]. An assessment of nanotoxic effects of nanoagrochemicals on seed germination and plant growth can be performed with the help of phytotoxicity assays and advanced microscopic techniques.

A plant or seed treated with nanomaterials may produce antimicrobial secondary metabolites as a defense against various plant pathogens. Plants can benefit from agronanochemicals to an extent because they can improve their overall disease resistance. There is a possibility that plants raised with nanomaterial-treated seeds will produce phytoestrogens. A phytoestrogen is a plant-produced bioactive secondary metabolite that plays an integral role in plant defenses and accumulates during times of stress or infection. This phytoestrogen is highly toxic to the human body as considered as endocrine disruptors. It interferes with hormone regulation in the reproductive system, leading to infertility and abnormal estrus cycles in women. In addition, there is little evidence that phytoestrogens have harmful effects on humans, but the use of risk assessment strategies to monitor crops raised from nanoagrochemical treated seeds is necessary before its consumption. As a safety precaution, phytoestrogens can be detected using a Sciex API III heated nebulizer atmospheric pressure chemical ionization interface coupled with tandem mass spectrometry.

It is well established that nanomaterials considerably impact the soil microbiome, including their abundance, diversity, and essential microbial processes, such as nitrogen fixation, mineralization, and plant growth-promoting activities [190, 191]. Studying the nanomaterial’s toxicity in soil and the influence of soil properties can also provide insight into the role of nanomaterials in soil pollution. An adverse effect of nanomaterials on soil microbiota can occur when they interact with the soil. The negative effects of most nanoparticles in soil are due to higher doses and mainly affect the enzymatic activities of soil microorganisms. Nanoparticles can inhibit critical steps in nutrient recycling such as ammonification, denitrification, nitrogen fixation, phosphate solubilization, and plant growth-promoting activities, all crucial for maintaining soil fertility and the ecosystem [192]. Zhang et al. [193] observed that silver nanoparticles changed the pH of soil and adversely affected soil microorganisms involved in nitrogen, carbon, and phosphorus cycles. Another study by Li et al. [194] found that soils amended with silver nanoparticles reduced seed development and caused silver bioaccumulation. Nanoparticles of other metallic metals, such as titanium and zinc, have been found to have adverse effects on the soil microbiota based on concentration and exposure duration [195, 196]. In a study by Rahman et al. [197], platinum nanoparticles stabilized with poly(vinylpyrrolidone) caused adverse effects on pea root microbiota, with a decrease in mycorrhizal fungi and rhizobia [198]. Using the soil metagenomic technique, it is possible to uncover the interactions and toxicity of different nanomaterials with the soil microbiome. In soil metagenomics, a nanomaterial-treated soil cultivation-independent molecular approach can be used to explore and exploit the immense diversity of soil microbial communities [191]. In this technology, soil microbiome DNA is isolated, and clone libraries are produced and screened. The nitrogen, carbohydrate, and phosphorus cycles are of high environmental importance, and nanomaterials appear to interfere with them. Nitrogen is fixed by urease, which converts urea into carbon dioxide and ammonia. Nanomaterials have been reported to reduce enzyme activity in several studies. An assessment of urease activity and its reduction in response to nanomaterials can also indicate reduced rhizospheric microbes [199].

It is possible for nanomaterial-based agrochemicals can seep into water bodies and enter the food chain (bioaccumulation) [40]. Many industries are interested in scaling up nanomaterials and nanomaterial-based agriproducts. Effluents from industrial plants contain nanomaterials that can contaminate water bodies. A nanomaterial may enter the body of an aquatic organism if it is disposed of in water and interfere with its physiology and feeding. A toxicology test can be used to evaluate the effects of nanomaterials or nanomaterial-based products on aquatic organisms. A toxicology test can be used to assess the potential for damage to aquatic environments and provide a database of information that can be used to assess the risk associated with using nanomaterials in a specific situation [42]. A common standard test species like fathead minnow (*Pimephales promelas*), the daphnids (*Daphnia magna, D. pulex, D. pulicaria and C. dubia*), the rainbow trout (*Oncorhynchus mykiss*), the sheepshead minnow (*Cyprinodon variegatu*), the zebra fish (*Danio rerio*), mysids are used commonly as a model organism to assess the risk of nanomaterials to examine the aquatic toxicity for in vivo studies [200–202]. Studying the genotoxicity effects and physiological responses of aquatic animals exposed to nanomaterials is possible through in vivo assays. Assays in vivo can provide information about hemocompatibility, immune response, histological profiling, protein analysis, and gene expression. In vitro assays (Cytotoxicity and cell viability assays) can also be used to determine the acute toxicity of nanomaterials on fish hepatoma and juvenile rainbow trout cell lines [203]. This approach will allow us to assess nanoagrochemical’s toxicity on aquatic organisms. Another novel, a new generation of nano-quantitative structure–activity/structure–property relationship tools for toxicity assessment, is examined. These tools are analyzed, including modeling methods and validation procedures, to evaluate whether these tools meet the current requirements for approving nanoformulations.

The applications of nanoagrochemicals also face major challenge due lack of fields data. The increasing reports on the applications of nanoagrochemicals have not encouraged the translation of laboratory data on field data. The reason can be the inadequate level of knowledge about nanoagrochemicals to enable reliable assessments of their risk. Several studies have investigated the fate of nanoagrochemicals, but hazard consequences cannot be determined adequately using protocols developed for other chemicals [204, 205]. Data insufficiencies result in some significant uncertainties related to environmental and consumer safety, essential to creating public confidence in products. It is paramount to answer several critical questions regarding the current state of uncertainty before being tried for field trials [205]. However, it is important to note that due to the uncertainty of regulatory frameworks as well as differing opinions globally, nanoagrochemical-based products for agricultural benefits are not flourishing and facing difficulties in reaching the market for field trials. The applications of nanoagrochemicals face major challenges due to emphasis on the sustainable development. Considering the United Nations Sustainable Development Goals 2030, it becomes increasingly important to use sustainable nanoagrochemicals in agricultural crop production to ensure compliance with regulation and consumer acceptance. The objective of sustainable nanoagrochemicals is to maximize their functional and economic performance while minimizing their adverse effects on the environment and human health [206]. Sustainable nanoagrochemicals (nanofertilizers, nanopesticides, nanoinsecticides etc.,) were prepared using green chemistry principles and encapsulated or coated by natural or biodegradable polymeric materials [207]. In order to achieve sustainability, it is crucial to integrate fundamental science (e.g., materials synthesis, characterization, and modeling) with engineering research (e.g., system design, fabrication, and testing) [208]. Hence, the application of sustainable nanoagrochemicals in the field cannot be judged based on a few studies, and to maximize their potential, sustainability nanoagrochemicals must be integrated into larger interdisciplinary research programs and/or government-funded research and development centers.

The successful application of nanotechnology in agro seed treatment products requires a comprehensive and effective strategy and coordinated risk management. It is important to consider the toxicological assessment of nanomaterials in seed treatment as a fundamental step toward identifying hazards related to applications of nanomaterials in the agro seed sector. A number of agronano products (Table 3) are now available on the commercial market [209], and efforts are being made worldwide to address and regulate the production and use of nanomaterials, either through legislation or by recommendations and guidance [76]. Governments and scientific organizations widely recognize the importance of nanomaterials-risk management in the agrochemical sector. Nanoagrochemicals can be considered a milestone in collaborative discussion, and information exchange forums are needed to ensure threat mitigation [210]. The combined efforts of governmental organizations, scientists, and social communities are required to prevent the adverse effects of nanoagrochemicals on humans and the environment. To successfully implement this nanotechnology within the profit margins for seed treatment, they must be capable of balancing system costs and benefits. For nanomaterials to be commercially useful, they must undergo extensive screening and optimization processes according to different seed types. A nanoscale seed treatment chemical must be developed with a simple handling process, low cost, sharp release system, and a high degradation rate. Seed treatments must overcome the major barriers to commercialization, poor demonstration of nanoscale products in field conditions, cost-effectiveness, consumer acceptance, and technical feasibility. A lack of public awareness campaign, inconsistency of the legal framework, and inconsistency of the regulatory framework affect the marketing of such nanoagrochemical products. Regulatory guidelines and frameworks are becoming increasingly important to resolve emerging issues associated with nanoscale agrochemicals. Nanoscale agrochemicals can undoubtedly alleviate many concerns caused by implementing routine agrochemicals, but more testing is necessary to lower agroecological risks. Innovative monitoring applications make it impossible for sustainable seed development and pollution control to be improved without creating nanoparticle contamination as a new hazard [211].

**Table 3:** An overview of the number of nanotechnology-enabled products, manufacturing firms, and countries involved in the plant-protection industry (Ref. [238] and https://product.statnano.com)

Type of nanoagrochemicals	Nanomaterial used	Nano-inspired products	Forming company	Key features
Nanofertilizer	Phosphorus and potassium	Fosvit K30	Kimitec Group, Spain	It is systemic and penetrates easily into plants, it facilitates potassium distribution
	Silver	Nano-Ag answer®	Urth Agriculture, USA	It helps plants and soil microorganisms survive and fix the organic matter
	Zinc	Nanozinc chelate fertilizer	Afme Trading Group, UK	It helps plants grow and develop by providing them with zinc metal element necessary for nutrient uptake by plants
	Zinc	Nanozinc	Alert Biotech, India	Zinc deficiency related crop issues are treated by this product
	Boron	Nanobor	Alert Biotech, India	The treatment is suitable for regions with high rainfall and acidic and sandy soils with poor organic matter
	Silica	Nanonutrients	NanoL and Baltic, Lithuania	By spraying it on leaf surfaces, all nutrients are delivered directly to chlorophyll and photosynthesis is enhanced
	Calcium	Nubiotek® Ultra Ca	Bioteksa, Mexico	It effectively helps crops endure climatic stress, pests, diseases, and aggressive conditions
	Iron and magnesium	Nubiotek® Hyper	Bioteksa, Mexico	The compound improves vegetative development by acting as an activator
	Titanium dioxide	Nanofertilizer	SilvertechKimya Sanayi veTicaret Ltd, Turkey	Plants grown from nano-TiO_2_ treated seeds showed significantly higher dry weight, photosynthetic rate, and chlorophyll-a formation
	Manganese molybdenum, zinc	Nanovec TSS 80	Laboratórios Bio-Médicin, Brazil	A defensive agent for seed treatment increases germination and leguminous plants’ emergence
	Potassium	Groagro 4: super kalium catalyst + T.E	Microwell Bio Solutions SdnBhd, Malaysia	Accelerating plant growth with improved nutritional value
	Potassium	Nano-potassium	Kanak Biotech	It helps plants develop roots and fruit, and improves their drought resistance
	Phosphorous	Nano-phosphorous	Kanak Biotech	Reduced tillering in cereals and poor seed development
	Urea	Nanourea (liquid) fertilizer	Indian Farmers Fertiliser Cooperative Limited	Nanourea contains nitrogen which effectively meets the crop’s nitrogen needs. Nanourea is more efficient than conventional urea in its use. It increases crop productivity, soil health, and nutritional quality
Nanopesticide	Silver	Nanosept® Aqua	Nanosept, Hungary	Seed treatments can benefit from its potential disinfectant properties
	Copper	Nanocu	BioNano Technology, Egypt	To improve root vigor while acting as an antifungal agent
	Silver	Blue lagoon UV-block	Dennerle, Germany	For the prevention of algal infection and growth in agriculture ponds
	Sulfur	Nanosulf foliar spray	Alert Biotech, India	Nanosulf is composed of elemental sulfur on nanosize particles, which dissolve completely in water. Nasosulf can also be used as a plant fertilizer, fungicide, and insecticide

## Conclusions and Future Perspective

Nanotechnology can be applied to nanoagrochemical delivery to improve the efficacy of seeds, reduce environmental pollution, support the sustainability of agricultural systems, and improve food security. By reducing the concentration of pesticides and fertilizers applied to the land, nanoagrochemical seed treatments increase the precision and effectiveness of seed protection products. In agro seed treatment, nanocarriers with controlled delivery can offer several advantages over conventional chemical delivery systems for sustainable agriculture, including biocompatibility, biosorption rate, low synthesis costs, thermo-plasticity, and ease of biodegradation. Developing nanoscale materials like nanocapsules, nanogels, nanofibers, nanoclay, and nanosuspensions can be used to design next-generation seed treatment strategies. More international and national risk assessment and management strategies need to be developed to achieve successful implementation. At last, we can conclude that using nanoscale products or formulations for agro seed treatments is essential for raising crops, and they have a pivotal role in sustainable crop production that cannot be ignored.

It is unclear what molecular mechanisms are responsible for the changes affecting seeds during and after treatment with nanoagroproducts. To understand the mechanism, further research is needed in the areas of genomic, proteomic, and metabolic to unreveal the effect of nanoformulations on seed responses. If we learn more about how nanoscale seed treatment works, we might be able to develop a viable seed processing technology. Future research should consider several factors to develop nanoagrochemicals for an advanced seed treatment; agrochemical parameters at the nanoscale should be optimized to achieve a reproducible and beneficial effect from seed treatment. Delivering the nanoagrochemicals to the right place and calculating the correct dose are challenging. When the parameters of nanoagrochemicals are optimized, it is possible to apply them to seeds so that significant treatment effects can occur. An appropriately responsive strategy and coordinated risk management are required to achieve nanotechnology’s positive effects in agro seed products. To identify the risks associated with nanomaterials used in seed treatment, conducting a toxicological assessment of those nanomaterials is crucial to answer their environmental issues. Research involving humans and eco-toxicology can help us understand how agroecosystems, nanoscale agrochemicals, exposure levels, and human beings interact. Nanoagrochemicals must be further investigated for agroecological toxicity and their mechanistic application.
